# 

**Published:** 1915-02-13

**Authors:** 


### CONTENTS.

PAGE

**The Telephone in Medical Practice** ... ... 431

**Mr. Samuel at the Poor-Law Conference** .. 432

**Hospital and Institutional News ...** ... ... 433

The Poor-Law Conference—An Appeal for Voluntary Residents—How to Raise Money from Abandoned Annual Events—An Emergency Surgical Service—Are the Voluntary Aid Detachments Abused ?—The Anomalous Position of the Workhouse Medical Officer—The Moral of the Epping Dispute—The Visitation of Patients in Mental Hospitals—The Value of Guardians' Visits—The New Out-patient Department at the Royal Free Hospital—Hospital Secretary's Fine Record—The Sanatorium Letter-writer—The Impatient Recruit—The Dual Appointment of Married Couples—The York Fever Hospital Extension Scheme—Modern Planning of Fever Hospitals—The New Pathological Department at Leicester—Middlesex Council and its Proposed Sanatorium—Higher Salaries at Birmingham Union Infirmary—"The Evolution of the Modern Hospital": A New Series—Medical Treatment and Poor-Law Disqualification—The Need for Beds after the War—Hospital Economy and Anæsthetics—Australia's Hospital Unit—This Week's Drug Market.

**A New Supply of Leeches ...** ... ... ... 439

The Medical Aspects of their Use.

**Medical Inventiveness in War** ... ... ... 440

Some Minor Examples of Ingenuity.

**Deaths Under Anæsthetics** ... ... ... 441

The Relation of Coroners and Hospital Staffs.

**Round the World** ... ... ... ... ... 442

Fellow-Workers and the Roll of Honour.

**Ethics of Giving** ... ... ... ... ... 443

The Ratio of Generosity to Income.

**Legal Notes for Institutional Workers** ... 444

Relation of Doctors to Insurance Committees. Miner's Nystagmus.

**Hospital Architecture and Construction** ... 445

General Hospital, Rangoon.

[*See over.*]

### CONTENTS—(*continued*).

| | PAGE |
|---|---|
| **Buildings Contemplated** ... ... ... ... | 446 |
| **Hospitals and Charitable Aid in New Zealand** | 447 |
| A Glance at State Aid in the Colonies. | |
| **Economy in the Generation and Use of Steam** | 448 |
| The Condensation of Steam. | |
| **The Editor's Letter-Box** ... ... ... ... | 449 |
| "By Belgians for Belgians." | |
| The Clinical Value of Poor-Law Infirmaries. | |
| **III.—Some Provincial Hospital Chapels** ... | 450 |
| Norfolk and Norwich Hospital. | |
| A Byzantine Chapel at Newcastle. | |
| A Remarkable Triptych at Nottingham. | |
| **Medical Appointments** ... ... ... ... | 452 |
| **Gifts and Bequests** ... ... ... ... ... ... | 452 |
| **Personalia** ... ... ... ... ... ... | 452 |
| **The Book Market** ... ... ... ... ... ... | 452 |
| **The Institutional Worker** ... ... (Suppl.) | 1 |
| Answer to January Question: Receiving Goods Without a Steward. | |
| A Division of Responsibility. | |
| Gas Grills. | |
| Question for February. | |
| Questions Invited. | |
| Employment and Training. | |
| Practical Matters. | |
| War Matters. | |

